# Survival with a Helping Hand: *Campylobacter* and Microbiota

**DOI:** 10.3389/fmicb.2015.01266

**Published:** 2015-11-09

**Authors:** Ivana Indikova, Tom J. Humphrey, Friederike Hilbert

**Affiliations:** ^1^Institute of Meat Hygiene, Meat Technology and Food Science, Department for Farm Animals and Veterinary Public Health, University of Veterinary Medicine, Vienna, Austria; ^2^Medical Microbiology and Infectious Diseases Group, College of Medicine, Swansea University, Swansea, UK

**Keywords:** mixed species, biofilm, protozoa, food, immunity

## Abstract

Campylobacteriosis is the most important bacterial food-borne disease in the developed world. Consumption of chicken meat, beef or raw milk, direct contact with ruminants and exposure to contaminated surface water or even consumption of tap water have been identified as risk factors for human disease. However, the most important risk factor is consumption of and/or handling contaminated chicken. *Campylobacter* spp. are fastidious microorganisms but must somehow survive outside the host, especially in food and agricultural environments and also resist the innate and humoral immune responses inside the host. In this paper we hypothesize that other microorganisms in mixed populations with *Campylobacter* may act to improve survival outside the host and may also protect the pathogen against the intestinal immune system. Our evidence for this hypothesis is based on: 1. newly generated microbial community analysis; 2. the prolonged survival of *Campylobacter* in mixed species biofilms and in co-culture with environmental bacteria; 3. improved survival in amoebae and rumen fluid; 4. sulfur release and iron uptake systems within the intestinal lumen. This would make *Campylobacter* an exceptional food-borne pathogen. With this in mind, new strategies are necessary to combat *Campylobacter* along the total food chain.

## Introduction

**FIGURE 1:**
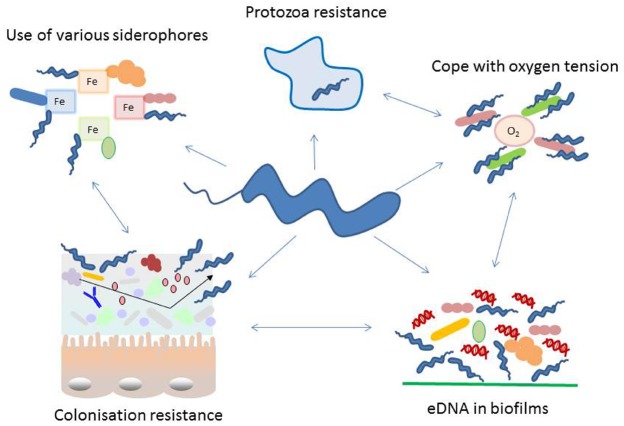
**Schematic representation of diverse adaptation mechanisms used by ***Campylobacter*** with special focus on interacting with other microorganisms.** *Campylobacter* interacts with its environment in numerous ways, many of these cooperate in an orchestrated manner. During colonization of a specific niche, the success is highly dependent on the microbial population residing at the surface. In the intestine colonization by *Campylobacter* is dependent on the intestinal microbiome. It influences attachment, replication, invasion, host immune response and colonization resistance. Several proteins exposed on the bacterial cell wall undergo phase variation, thus changing their antigenic properties. Recently extracellular DNA (eDNA) has been described in *Campylobacter* biofilms and these may not only be used in this natural competent bacterium to modify its genome but as well for signaling and biofilm degradation. Moreover, *Campylobacter* has evolved features to facilitate the survival with a limited genome, like phase variation and the use of essential nutrients produced by other microorganisms. Such capability includes the co-existence with aerobic bacteria to reduce the toxic effect of oxygen, survival within protozoa and resistance to their digestive metabolism and smart methods to acquire iron and sulfur from the microenvironment.

*Campylobacter jejuni* (*C. jejuni*) is the most important bacterium causing foodborne infections in the developed world. Infection with this pathogen leads to severe economic loss in industrial countries and it is estimated that 1% of the European population is infected per year (Humphrey et al., 2014). The European Food Safety Authority (EFSA) reported 214268 confirmed human cases (with 31 death occurring) due to campylobacteriosis compared to 91034 human cases of salmonellosis in 2012 in Europe (EFSA and European Centre for Disease Prevention and Control, 2014). These figures are likely to be a significant under-estimation. In the USA, the Centers for Disease Control and Prevention (CDC) estimates that there are 1.3 million cases per year and found a 2% increase in cases from 2010 to 2013. In contrast, a decrease in *Salmonella* infections of 7% in the same period has been reported (CDC, 2014). Most of these *Campylobacter* infections are sporadic. Outbreaks, when a group of individuals is affected, have been primarily traced back to raw or incompletely pasteurized milk and water (Palmer et al., 1983; Evans et al., 1996; Fernandes et al., 2015). However in most sporadic infections consumption of chicken meat accounts for most human cases but beef, raw or incompletely pasteurized milk and contaminated water contribute to the high numbers of reported cases of disease. *C. jejuni*, the most important species causing human disease, can reside in the intestine of most warm-blooded animals, sometimes with distinct effects on the host such as severe disease symptoms, inflammation of gut mucosa and even penetration into deeper tissues by epithelial cell invasion. This major foodborne pathogen can also colonize the gut of animals without almost any symptoms of disease. This paradox is still not well understood but a number of recent publications point out that other microorganisms of the gut microbiome may have a substantial impact on the colonization ability of *Campylobacter* and the development of disease symptoms in the animal and human host. Many pathogens, including *Campylobacter* need sulfur and iron as essential micronutrients. To acquire these nutrients *Campylobacter* has some effective mechanisms to take up iron from diverse bacterial siderophores and to deplete sulfur from host cells (Naikare et al., 2013; Zeng et al., 2013; Vorwerk et al., 2014). As *Campylobacter* are well-adapted intestinal microorganism, these bacteria can persist in the rumen of bovine hosts (Stanley et al., 1998; Heugas, 2015). Moreover, as a foodborne pathogen it is still puzzling how *Campylobacter* can survive outside animal hosts on chicken meat, or in environmental water. *C. jejuni* is able to survive in harsh natural environments with the help of other microorganisms. Moreover resistance to digestion by amoeba may help its survival in water (Bui et al., 2012) and oxygen reducing environmental microorganisms may support its survival under atmospheric oxygen tension (Hilbert et al., 2010; Bui et al., 2011). This leads to the assumption that *Campylobacter* benefits by interacting with different microorganisms. In this review we give special attention to these interactions and hypothesize that several of these are important survival features in- and outside the host (Figure 1).

## Microbial Community and *Campylobacter* Infection or Colonization

As *C. jejuni* pathogenicity in humans is not well understood, researchers have been keen to find an animal model to study disease symptoms and virulence. This was hampered by a so-called colonization resistance in the mouse model, until Bereswill et al. (2011) established a mouse model by depleting the gut microbiome with a cocktail of antibiotics. In this model, mice orally infected with *C. jejuni* suffer from enterocolitis, diarrhea and show a humoral immune response similar to that seen in human campylobacteriosis (Bereswill et al., 2011). Additionally, previous inflammation of the intestine caused by *Toxoplasma gondii* has been shown to substantially enhance colonization of *C. jejuni* of the total gastro-intestinal tract (GT), leading to bacteraemia and infiltration of spleen and liver in the mouse model (Haag et al., 2012). In mice the colonization of the GT tract with *C. jejuni* is accompanied by high intestinal loads of commensal *Escherichia coli* (Haag et al., 2012). Furthermore microbial community studies revealed in animals and humans likewise a change in the intestinal microbiome when colonized with *Campylobacter*. *C. jejuni* colonization in chickens is associated with lower numbers of *Lactobacillus* and *Corynebacterium* species but numbers of *Streptococcus* and *Ruminococcaceae* are higher (Kaakoush et al., 2014). Humans with campylobacteriosis found to have a higher abundance of *Bacteroidetes* and *Escherichia* spp. (Dicksved et al., 2014). In a recent study the gut microbiota of poultry abattoir workers was analyzed using metagenomics. The researchers showed that *Campylobacter* colonization of these abattoir workers was associated with a long-term change of their microbiome (Dicksved et al., 2014).

Promising strategies for the reduction of human disease are the use of probiotics for competitive exclusion of *Campylobacter* colonization in broilers. Especially *Lactobacillus* spp. and *Bifidobacteria* have been shown to be successful in reducing *Campylobacter* colonization in broilers (Baffoni et al., 2012; Ganan et al., 2013; Tareb et al., 2013; Cean et al., 2015). A very recent study was able to identify that cell-surface aggregation-promoting factor 1 of *Lactobacillus gasseri* LG2055 is relevant for competitive exclusion of *C. jejuni* 81–176 (Nishiyama et al., 2015). Stimulation of the immune defenses (activation of interleucines and defensins) and the modulation of epithelial cell barrier integrity was also implicated as activity in probiotic bacteria against *Campylobacter* spp. (Messaoudi et al., 2012). Nevertheless, complete prevention of colonization of broilers with *Campylobacter* using probiotics has not been successful so far (Hermans et al., 2011). The colonization ability of *Campylobacter* is dependent on the microbiome and also these bacteria change the microbiome of a colonized/infected host. We hypothesize that these changes are linked to host immune responses that allow *Campylobacter* colonization with either no signs of disease or severe symptoms.

## Survival Strategies in *Campylobacter* Biofilms

The most common lifestyle of bacteria on surfaces in natural environments or artificial niches, such as food processing equipment, is growth in biofilms or bioaggregates. These microbial structures are bound together by an extracellular polymeric matrix (EPM). The EPM is a complex mixture of various polysaccharides (PSs), proteins and nucleic acids. Diverse PSs have different functions in biofilm formation, such as stability, biofilm adherence, uptake and maintaining of nutrients, and resistance to various stressors (Limoli et al., 2015). Different proteins of the EPM are responsible for the EPM adhesion properties to biotic or abiotic surfaces and others are likely to play a role in synthesis, modification, stabilization and degradation of the EPM (Latasa et al., 2006; Fong and Yildiz, 2015). Extracellular DNA (eDNA) has been shown recently to be an abundant component of various mono-species and multi-species biofilms. The presence of eDNA is important for surface attachment, biofilm growth and might be a source of horizontal gene transfer but provides nutrition during oligotrophic conditions as well (Finkel and Kolter, 2001; Steinberger et al., 2002; Whitchurch et al., 2002; Molin and Tolker-Nielsen, 2003; Qin et al., 2007). In *C. jejuni* biofilms, eDNA was detected both in the supernatant and surrounding the biomass (Brown et al., 2015). The importance of eDNA for biofilm formation can be seen in *C. jejuni* RM1221, a variant unable to form biofilms. This strain carries three different copies of DNase I, which are constantly released into the environment and cleave eDNA. Inoculating a biofilm of *C. jejuni* NCTC 11168 with a growing culture of RM1221 led to a rapid and complete removal of the mature biofilm without compromising cell viability (Brown et al., 2015). Additionally, biofilm formation is based on quorum sensing (QS), which is a population-dependent signaling mechanism, which involves synthesis, secretion and detection of signaling molecules called autoinducers (Bassler, 1999). By sensing these autoinducers bacterial communities can initiate and regulate their response to the signal. In many Gram-negative bacteria QS is based upon homoserine lactone (HSL). This molecule was revealed as controlling the expression of numerous traits including bioluminescence, antibiotic, and virulence factor production. Numerous HSLs are involved in biofilm differentiation and control the expression of extracellular virulence factors (Pearson et al., 1994; Davies et al., 1998). For interspecies communication both Gram-positive and Gram-negative bacteria, share common sensing systems involving autoinducer-2 (AI-2; Bassler, 1999). A precursor of AI-2 is produced by *luxS*, first described in *Vibrio harveyi* (Bassler et al., 1997). The description of a *luxS* ortholog (Cj1198) in *C. jejuni* suggests its involvement in regulation of motility (Parkhill et al., 2000; Elvers and Park, 2002). Despite much research on the function of AI-2 in *C. jejuni*, no receptor molecule for it has yet been discovered in this bacterium.

## Reduction of Oxygen Level, a Driving Force in *Campylobacter* Survival in- and Outside Hosts

*Campylobacter jejuni* requires oxygen but cannot grow under atmospheric oxygen tension as it is a microaerophilic bacterium. Despite sensitivity to high oxygen tensions *in vitro*, viable and culturable *Campylobacter* can be isolated from food surfaces. Mechanisms, by which *Campylobacter* survives, can rely on interspecies interaction with Pseudomonadaceae, like growth in biofilms. Hilbert et al. (2010) showed that different *C. jejuni* strains: food, human and environmental isolates, showed prolonged survival under atmospheric oxygen tension in co-culture with type strains and isolates of different Pseudomonadaceae. Similarly, mixed biofilms of *Campylobacter* and *Pseudomonas aeruginosa* are able to enhance the viability and culturability of the former under atmospheric oxygen tension (Ica et al., 2012).

Oxygen levels are the main driving forces in construction of oral biofilms. Oxygen pressure has not been thought of as an important driving force in the intestine, until accurate measurements of levels of this compound were conducted within the different parts of the gut. This showed that most of the gut was microaerobic with only a few areas being strictly anaerobic (Albenberg et al., 2014). Studies on different bacteria in regards to the composition of mixed biofilm communities are well studied in oral and dental biofilms (Jenkinson and Lamont, 2005). A first attachment of aerobic bacteria paves the way for a microaerobic flora, including certain oral *Campylobacter* species, important pathogens of the oral cavity, and finally anaerobic bacteria follow microaerobic species in biofilm construction. Similar community successions may also occur in the gut. Our recent studies on *C. jejuni* in co-culture with *Clostridium perfringens* or *Cl. difficile* under microaerobic atmosphere *in vitro* have shown growth of both important intestinal *Clostridia* species within mixed biofilms with *Campylobacter* (Hilbert et al., 2014). If this is also true for the main location of *Campylobacter* colonization in the intestine, this could explain the change in the gut microbiome of mice, chicken and humans (Haag et al., 2012; Dicksved et al., 2014; Sofka et al., 2015). The formation of mixed biofilms has been described as a mechanism to avoid the immune response of the host and can lead to persistent infection in *Staphylococcus* (Thurlow et al., 2011; Scherr et al., 2014) We speculate that this can be the case for *Campylobacter* colonization as well. Thus, multi-species biofilms may allow *Campylobacter* to be masked against the host immune system and furthermore this could explain high intestinal loads of *Campylobacter* without clinical signs of disease.

## *Campylobacter jejuni* is an Amoeba-Resistant Bacterium and can Survive in Rumen Fluid

It has been shown that resistance to digestion by amoeba may help *Campylobacter* to survive in environments like water (Axelsson-Olsson et al., 2010; Bui et al., 2012). In the vegetative cycle, water protozoa live primarily by phagocytosis of bacteria. If bacteria survive within protozoa, they can be protected until lysis of the protozoa host. Thus, amoebae represent an important reservoir of bacteria in the environment. Internalized bacteria are not only protected from undesirable environmental stressors like chlorine, bactericides and antibiotic residues but could possibly replicate in protozoa. Additionally we hypothesize that these resistant bacteria might parasitize on essential nutrients, presented by amoeba digestion and speculate that digested bacteria might provide an important nutrient source for amoeba-resistant bacteria (Kebbi-Beghdadi and Greub, 2014). *C. jejuni* can actively invade *Acanthamoeba polyphaga* and persist and replicate in vacuoles (Olofsson et al., 2013). Additionally, *Campylobacter* has been shown in ciliates in the drinking water and *in vitro* can effectively survive in *Tetrahymena pyriformis* and *Acanthamoeba castellanii* (Snelling et al., 2005).

Fecal shedding of *C. jejuni* in cattle has been described to be dependent on drinking water, feeding and the presence of other animals (Ellis-Iversen et al., 2009). Different associations with *Campylobacter* colonization have been revealed in dairy cows versus calves (Grove-White et al., 2010; Klein et al., 2013). A possible explanation could be that the rumen is not yet developed in young calves. The anaerobic basic condition of the bovine rumen is not at all suitable for survival or growth, although, it has been shown to be the only natural location of *C. jejuni* next to the small intestine of the bovine host (Stanley et al., 1998). Ruminants depend on the microbial ecosystem of the fore stomachs to effectively digest carbon rich plant components. The ruminal flora and fauna form an ecological unit with the host animal. Very recently we were able to show that *C. jejuni* is able to survive over an extended period of time in rumen fluid with viable protozoa. In contrast, sterile rumen fluid without protozoa was a rather unfavorable condition and *Campylobacter* was not able to survive in it (Heugas, 2015).

## Sulfur and Iron Release from Host Cells

*Campylobacter jejuni* is unable to utilize sugar but relies on amino acids such as aspartate, glutamate, serine, proline, and organic acids for its energy metabolism (Hofreuter et al., 2008). Next to accessing energy sources, gaining micronutrients like metals, which are often limited, is demanding. Most important are iron and sulfur as key elements for many enzymes. Within the host intestine *Campylobacter* can release cysteine containing peptides from epithelial host cells to access sulfur for its metabolism (Vorwerk et al., 2014). This release of sulfur in the intestinal lumen might lead to a change in the intestinal environment as the element has been shown to have an influence on the intestinal pH and alters inflammatory mediators (Kerr et al., 2011). This release of sulfur caused by *Campylobacter* might, in part, be responsible for the described changes in the composition of the intestinal microbiome by 1. a change in pH and 2. a shift toward sulfur-requiring bacteria.

Iron uptake is essential for *Campylobacter* growth (Palyada et al., 2004). Many crucial metabolic enzymes in *Campylobacter* depend on a functional sulfur–iron complex (Stahl et al., 2012). Most important iron sources are bound in complexes and are not available as free iron. In order to grow in low iron environments such as within the host, most microorganisms produce and release siderophores to bind iron and have specific transporters to take them up again. These siderophores, metal-chelating agents released in the environment, can capture iron in the form of Fe^3+^ and this complex is transported through the membrane (outer membrane) to the cytosol or periplasmic space. *Campylobacter* are not able to synthetize their own siderophores for its iron metabolism, but are able to use ones released by other bacteria and take up these ferric complexes. *Campylobacter* is not dependent on one specific siderophore produced by a certain bacterial species but is able to use different kinds of siderophores (ferric-enterobactin, hemin, ferric transferrin, and lactoferrin) by using distinct transporters (Palyada et al., 2004; Ridley et al., 2006; Miller et al., 2008; Xu et al., 2010; Stahl et al., 2012). It is known that the release of siderophores can change the microbiome of an environmental niche but these bacterial–bacterial interactions have not been analyzed with regard to which bacteria can benefit.

## Conclusion

Despite campylobacteriosis being the most important bacterial foodborne disease in the developed world there is limited success in strategies to combat this disease. Herein we highlight a so far underestimated perspective of this pathogen where it takes advantage of other microorganisms. Assembling information in the context of *Campylobacter* survival in the intestinal microbiome, in mixed bacterial biofilms, in gaining micronutrients from other microorganisms and last but not least in hiding in and acquiring essential nutrition from amoeba, we conclude that *Campylobacter* may be more than a single pathogenic species but relies in many ways on other bacteria. Given that most strategies to reduce campylobacteriosis have not been successful new thinking in regards to microbial–microbial interactions and thus different control strategies are crucial to fight this major zoonotic pathogen.

### Conflict of Interest Statement

The authors declare that the research was conducted in the absence of any commercial or financial relationships that could be construed as a potential conflict of interest.
